# Foreign Body in Root Canals of Two Adjacent Deciduous Molars: A Case Report

**DOI:** 10.5005/jp-journals-10005-1184

**Published:** 2013-04-26

**Authors:** Kanika Singh Dhull, Sonu Acharya, Prayas Ray, Rachita Singh Dhull

**Affiliations:** Senior Lecturer, Department of Pedodontics and Preventive Dentistry Kalinga Institute of Dental Sciences, Bhubaneshwar, Odisha, India; Reader, Department of Pedodontics and Preventive Dentistry Kalinga Institute of Dental Sciences, Bhubaneshwar, Odisha, India; Assistant Professor, Department of Pedodontics and Preventive Dentistry, SCB Dental College, Cuttack, Odisha, India; Attending Consultant, Department of Pediatrics, Fortis Escorts Heart Institute, New Delhi, India

**Keywords:** Foreign body, Deciduous molars, Root canals

## Abstract

Children often tend to have the habit of inserting foreign objects in the oral cavity unknowingly for relief of dental pain. Sometimes, children do not reveal to their parents due to fear. These foreign objects may act as a potent source of infection and painful condition. The discovery of foreign bodies in the teeth is a special situation, which is often diagnosed accidentally. Detailed case history, clinical and radiographic examinations are necessary to come to a conclusion about the nature, size, location of the foreign body and the difficulty involved in its retrieval. Here is a case report, where foreign object was accidentally lodged in the carious deciduous molars by a child.

**How to cite this article:** Dhull KS, Acharya S, Ray P, Dhull RS. Foreign Body in Root Canals of Two Adjacent Deciduous Molars: A Case Report. Int J Clin Pediatr Dent 2013;6(1):38-39.

## INTRODUCTION

Foreign bodies inside tooth are diagnosed accidentally on clinical or radiographic examination of the tooth which may be associated with infection, pain, swelling and recurrent abscesses as a sequelae to the pulpal exposure and lodgment of the foreign body.^1^ The presence of foreign objects retrieved from the root canals and pulp chambers of the permanent teeth have been reported,^2^ the presence of foreign objects found in the deciduous teeth is an uncommon situation.^3^

## CASE REPORT

**Fig. 1:**
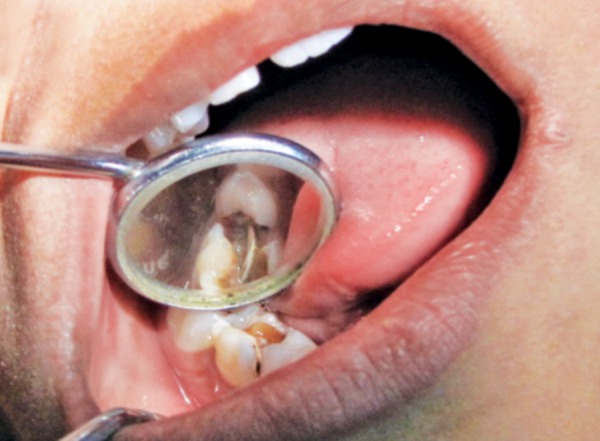
Clinical picture showing the lodged foreign object in

**Fig. 2:**
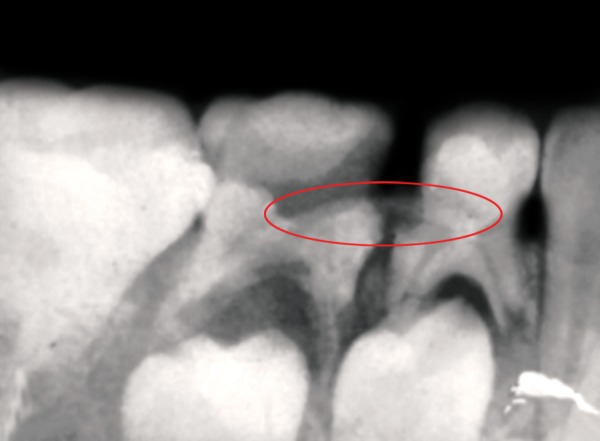
IOPA radiograph showing radiopaque object in the canals of adjacent deciduous molars

An 8-year-old girl reported to the Department of Pedodontics and Preventive Dentistry, Institute of Dental Sciences, Bhubaneswar, Odisha, India, with a chief complaint of pain in the deciduous right mandibular first molar tooth (Fig. 1). Patient gave a history of pain from the past 1 month. The patient was quite apprehensive and did not allow for clinical examination, so was sent for intraoral periapical (IOPA) radiographs. Radiographic examination of the tooth revealed a radiopaque object overlapping the image of the tooth and a furcal abscess (Fig. 2). Same side lingual opposite side buccal (SLOB) rule was performed to confirm whether the foreign object was lying in the root or in the adjacent soft tissues. It was observed that the pin was lodged in the distal canal of primary mandibular first molar and distal canal of primary mandibular second molar (Fig. 2).

**Fig. 3:**
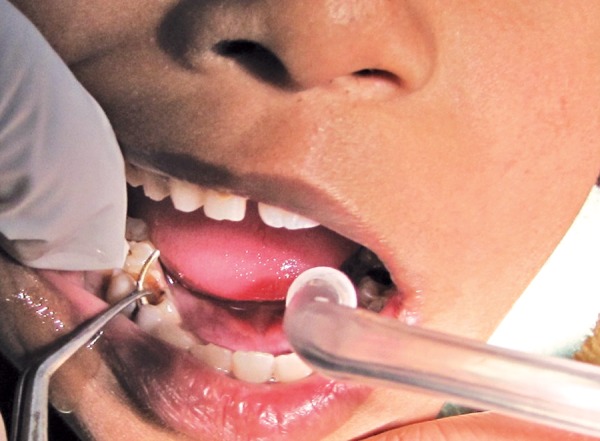
Pin being removed with the tweezer

**Fig. 4:**
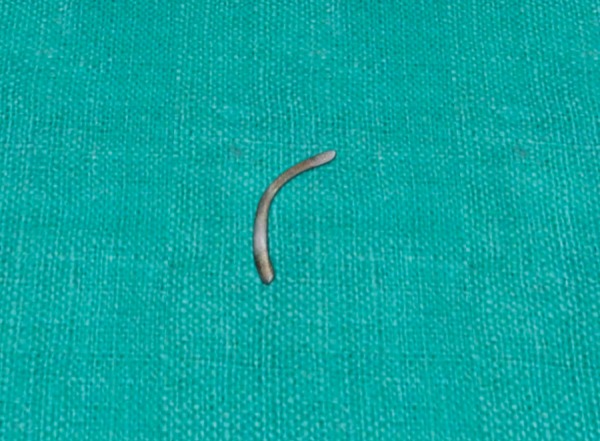
Pin after removal

An ultrasonic scaler was used to clear the debris from the root canal orifices and also to facilitate loosening of the pin. When the ball pin was adequately visible clinically, it was engaged with a tweezer and removed (Figs 3 and Figs 4). Since the teeth were nonrestorable, so decision was made to extract the teeth under local anesthesia.

## DISCUSSION

Frequently, foreign bodies are found in the oral and nasal cavities of children and are discovered by the dentist during routine examinations. These objects may be the result of the child's own action and may cause pain, edema and tooth fracture. Different types of foreign objects were reported to be lodged in the root canals and the pulp chamber, which ranged from pencil leads,^4^ darning needles,^2^ metal screws,^5^ to beads^6^ and stapler pins.^7^ Grossman^8^ reported retrieval of indelible ink pencil tips, brads, a tooth pick, adsorbent points.^8^ A radiograph can be of diagnostic significance especially if the foreign body is radiopaque. McAuliffe^7^summarized various radiographic methods to be followed to localize a radiopaque foreign object as parallax views, vertex occlusal views, triangulation techniques, stereo-radiography and tomography. Vertex occlusal view is no longer favored because of relatively high radiation exposure to the lens of the eye and because the primary beam is aimed toward the abdomen. Removal of foreign objects from the root canal is often a very difficult procedure. The procedure is even more complicated, if the foreign body is unusual.

Though, the presence of foreign objects retrieved from the root canals and pulp chambers of the permanent teeth have been reported, the presence of foreign objects found in the deciduous teeth is an uncommon situation. This case discusses the presence of foreign object in the root canals involving two deciduous teeth. Timely diagnosis and management of foreign object embedded in the tooth should be done to avoid further complications like ingestion,^9^chronic maxillary sinusitis if embedded in upper teeth.^10^

## CONCLUSION

The above case report discusses the management of teeth with impacted foreign objects in the tooth. The pin was embedded in two adjacent mandibular deciduous molars which is unusual. There is a definite need for a proper classification of foreign bodies in and around the teeth and a treatment algorithm to be followed in such clinical situations.
